# Supplementary material for: Identification of Freezing-Responsive microRNAs and Their Targets in Chinese Jujube by Small RNA and Degradome Sequencing
Source: Int J Mol Sci. 2026 Sep 4;27(17):7912. doi: 10.3390/ijms27177912 (PMC13566890; doi:10.3390/ijms27177912)
Supplement: Supplementary file 1 [file ijms-27-07912-s001.zip › Supplementary Material S2/novel_miR_59.pdf]

Provisional ID	:	novel_miR_59
Score total	:	0.5
Score for star read(s)	:	-1.3
Score for read counts	:	0
Score for mfe	:	0.2
Score for randfold	:	1.6
Score for cons. seed	:	
Total read count	:	1732
Mature read count	:	1655
Loop read count	:	1
Star read count	:	76

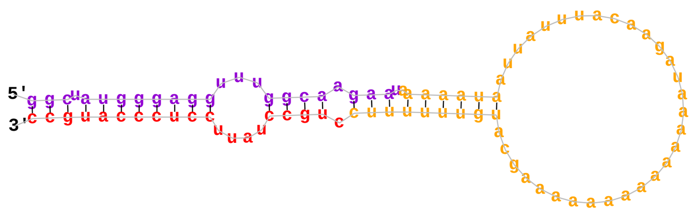

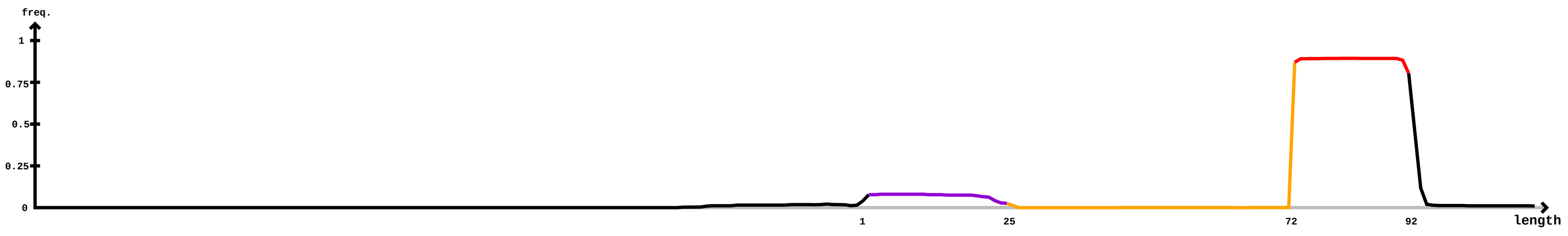

Star Mature

```
5'- auaaccaucaaaagugaagacccaaugcauuaaauuaaucaauagugucuccuucuuuggacagcucccucuuaucgucauuuucaaggcugacaucaacgcguuuuaaagagagauaaagaacuuauggaaauuuuuggcuaugggagguuuggcaagaauaaaaauaauuauuuacaagauaaaaaaaaaaaaaaagcauguuuuuuccugccuauuccucccaugccaaugauuuccuuuuguuccucu -3'  obs
    auaaccaucaaaagugaagacccaaugcauuaaauuaaucaauagugucuccuucuuuggacagcucccucuuaucgucauuuucaaggcugacaucaacgcguuuuaaagagagauaaagaacuuauggaaauuuuuggcuaugggagguuuggcaagaauaaaaauaauuauuuacaagauaaaaaaaaaaaaaaagcauguuuuuuccugccuauuccucccaugccaaugauuuccuuuuguuccucu      exp
    ...........................................((((((..((((((((..((.......(((((.......))).))......))..))))))))))))))..((((..(.(((((((.(((((.((((((((...((((.(((..((((((............................)))))))))).))))....)))))))))))).))))))).)..))))....
```

read	reads	mm	sample
aaagagagauaaagaacuuauggaa	1	0	JT-2
aaagagagauaaagaacuuauggaa	2	0	JT-3
aaagagagauaaagaacuuauggaa	2	0	LT-2
aagagagauaaagaacuuauggaaa	1	0	LT-2
gagagauaaagaacuuCuggaa	1	1	LT-3
agagauaaagaacuuaug	1	0	LT-1
agagauaaagaacuuauggaaau	1	0	LT-1
agagauaaagaacuuauggaaauu	1	0	JCK-3
agagauaaagaacuuauggaaauu	1	0	JCK-2
agagauaaagaacuuauggaaauu	1	0	JCK-1
agagauaaagaacuuauggaaauuuu	1	0	JCK-1
agagauaaagaacuuauggaaauuuu	1	0	JCK-2
agagauaaagaacuuauggaaauuuu	3	0	JCK-3
gagauaaagaacuuauggaaauuuu	2	0	JCK-1
gagauaaagaacuuauggaaauuuuu	1	0	JCK-1
gagauaaagaacuuauggaaauuuuuggcu	1	0	JCK-3
uaaagaacuuauggaaauu	7	0	LCK-3
uauggaaauuuuuggcuaugga	2	0	JCK-3
uauggaaauuuuuggcuaugga	3	0	JCK-2
aaauuuuuggcuaugggaggu	1	0	LT-3
aaauuuuuggcuaugggagguu	1	0	LT-3
aauuuuuggcuaugggaggu	3	0	LT-1
aauuuuuggcuaugggagguuuggc	3	0	LT-2
uuggcuaugggagguuuggc	4	0	LT-2
Cuggcuaugggagguuuggcaag	1	1	JCK-3
uggcuaugggagguuuggca	1	0	LCK-3
uggcuaugggagguuuggca	2	0	LT-1
uggcuaugggagguuuggca	1	0	JCK-3
uggcuaugggagguuuggca	2	0	JCK-1
uggcuaugggagguuuggca	1	0	JCK-2
uggcuaugggagguuuggcaa	3	0	LT-3
uggcuaugggagguuuggcaa	2	0	JCK-3
uggcuaugggagguuuggcaag	2	0	LT-3

Star Mature

auaaccaucaaaagugaagacccaaugcauuaaauuaaucaauagugucuccuucuuuggacagcuccucuuaucgucauuuucaaggcugacaucaacgcguuuuaaagagagauaaagaacuuauggaaauuuuuggcuaugggagguuuggcaagaauaaaaauaauuauuuacaagauaaaaaaaaaaaaaagcauguuuuuuccugccuauuccucccaugccaaugauuuccuuuuguuccucu

uggcuaugggagguuuggcaag	1	0	JCK-1
uggcuaugggagguuuggcaag	5	0	JCK-3
uggcuaugggagguuuggcaag	2	0	JCK-2
uggcuaugggagguuuggcaag	13	0	JT-1
uggcuaugggagguuuggcaaga	2	0	LT-3
uggcuaugggagguuuggcaaga	4	0	JCK-2
uggcuaugggagguuuggcaaga	3	0	JCK-3
uggcuaugggagguuuggcaaga	3	0	JCK-1
uggcuaugggagguuuggcaaga	6	0	JT-1
uggcuaugggagguuuggcaagaUu	2	1	JCK-1
ggcuaugggagguuuggca	3	0	LCK-3
ggcuaugggagguuuggcaag	14	0	JT-1
ggcuaugggagguuuggcaag	1	0	JCK-3
ggcuaugggagguuuggcaaga	3	0	JCK-1
ggcuaugggagguuuggcaaga	1	0	JCK-3
ggcuaugggagguuuggcaaga	2	0	LT-3
ggcuaugggagguuuggcaaga	1	0	JCK-2
ggcuaugggagguuuggcaagaUu	17	1	LCK-2
ggcuaugggagguuuggcaagaUu	25	1	LCK-1
ggcuaugggagguuuggcaagaauU	2	1	JCK-3
ggcuaugggagguuuggcaagaauU	1	1	JCK-2
cuaugggagguuuggcaagaa	2	0	JCK-3
cuaugggagguuuggcaagaa	3	0	JCK-2
augggagguuuggcaagaUu	1	1	JCK-3
Cauaaaaaaaaaaaaaagc	1	1	LT-3
uuuuuuccugccuauucc	1	0	LT-3
ccugccuauuccucccaugccaaug	1	0	LT-3
cugccuauuccucccaug	2	0	JCK-3
cugccuauuccucccauU	1	1	LT-3
cugccuauuccucccaug	1	0	LT-3
cugccuauuccucccaug	13	0	LCK-3
cugccuauuccucccaug	3	0	JCK-2
cugccuauuccucccaugc	24	0	LT-3
cugccuauuccucccaugc	7	0	JT-1
cugccuauuccucccaugc	6	0	JCK-2
cugccuauuccucccaugc	25	0	LT-1
cugccuauuccucccaugc	2	0	JCK-3
cugccuauucUucccaugc	12	1	LCK-3
cugccuauuccucccaugc	40	0	LCK-3
cugccuauuccucccaugc	8	0	LT-2
Uugccuauuccucccaugc	1	1	LT-1
cugccuCuuccucccaugc	1	1	LT-3
cugccuauuccucccaugU	4	1	LCK-3
cugccuCuuccucccaugc	1	1	LCK-3
cugccuauuccucccaugc	6	0	JCK-1
cugccuauuccucccaugA	1	1	LT-1
cugccuauuccucccaugcc	31	0	JCK-3
cugccuauuccucccaugcU	8	1	JCK-2
cugccuauuccucccaugcc	8	0	JT-3
cugccuauuccucccaugcA	2	1	LT-3
cugccuauuccucccaugcc	2	0	JT-2
cugccuauuccucccaugcU	6	1	LT-1
cugccuauuccucccaugcc	73	0	LT-2
cugccuauuccucccaugcU	2	1	LT-2
cugccuauuccucccaugcU	5	1	LCK-3
cugccuauuccucccaugcc	121	0	LT-3
cugccuauuccucccaugcA	2	1	LT-2
cugccuauuccucccaugcc	53	0	JT-1
cugccuauuccuUccaugcc	2	1	JCK-3
cugccuauuccucccaugcc	160	0	LCK-3
cugccuauuccucccaugcU	6	1	LT-3
cugccuauuccucccaugcU	1	1	JCK-3
cuNccuauuccucccaugcc	1	1	LT-2
cugccuauuccucccaugcc	100	0	LT-1
cugccuauuccucccaugcc	31	0	JCK-1
cugccuauuccucccaugcU	2	1	JCK-1
Uugccuauuccucccaugcc	1	1	LT-3
cugccuCuuccucccaugcc	1	1	LCK-3
cugccuauuccucccaugcc	46	0	JCK-2
cugccuauuccucccaugUc	1	1	LT-1

```
                                                                                                                                         Star                                                                    Mature
auaaccaucaaaagugaagacccaaugcauuaaauuaaucaauagugucuccuucuuuggacagcuccucuuaucgucauuuucaaggcugacaucaacgcguuuuaaagagagauaaagaacuuauggaaauuuuuggcuaugggagguuuggcaagaauaaaaauaauuauuuacaagauaaaaaaaaaaaaaaagcauguuuuuuccugccuauuccucccaugccaaugauuuccuuuuguuccucu
.........................................................................................................................................................................................................cugccuCuuccucccaugcca.....................  1    1    LCK-3
.........................................................................................................................................................................................................Uugccuauuccucccaugcca.....................  2    1    JCK-1
.........................................................................................................................................................................................................cugccuauuccucccaugcca.....................  33   0    LT-2
.........................................................................................................................................................................................................cugccuauuccucccaugcca.....................  94   0    JCK-2
.........................................................................................................................................................................................................cugccuauuccucccaugccU.....................  1    1    LT-3
.........................................................................................................................................................................................................cugccuauuccucccaugcca.....................  61   0    JCK-3
.........................................................................................................................................................................................................cugccuCuuccucccaugcca.....................  1    1    JCK-2
.........................................................................................................................................................................................................cugccuauuccucccaugcca.....................  46   0    JCK-1
.........................................................................................................................................................................................................cugccuauuccucccaugcca.....................  66   0    LT-1
.........................................................................................................................................................................................................Uugccuauuccucccaugcca.....................  1    1    JCK-3
.........................................................................................................................................................................................................cugccuauuccucccaugcca.....................  44   0    LCK-1
.........................................................................................................................................................................................................cugccuauuccucccaugcca.....................  11   0    JT-1
.........................................................................................................................................................................................................cugccuauuccucccaugcca.....................  57   0    LCK-3
.........................................................................................................................................................................................................cugccuauuccNcccaugcca.....................  1    1    JCK-1
.........................................................................................................................................................................................................cugccuauuccucccaugcca.....................  9    0    JT-3
.........................................................................................................................................................................................................cugccuauuccucccaugcca.....................  68   0    LCK-2
.........................................................................................................................................................................................................cugccuauuccucccaugcca.....................  1    0    JT-2
.........................................................................................................................................................................................................cugccuauuccucccaugccU.....................  1    1    LT-1
.........................................................................................................................................................................................................cugccuauuccucccaugcca.....................  81   0    LT-3
.........................................................................................................................................................................................................cugccuauuccucNcaugcca.....................  1    1    LCK-1
.........................................................................................................................................................................................................cugccuCuuccucccaugcca.....................  1    1    JCK-1
.........................................................................................................................................................................................................cugccuauuccucccaugccC.....................  1    1    LCK-3
.........................................................................................................................................................................................................cugccuauuccucccaugccC.....................  1    1    LT-1
.........................................................................................................................................................................................................cugccuauuUcucccaugccaa....................  1    1    JCK-3
.........................................................................................................................................................................................................cugccuauuccucccaugccaa....................  53   0    JCK-3
.........................................................................................................................................................................................................cugccuauuccucccaugccaa....................  2    0    LT-2
.........................................................................................................................................................................................................cugccuauuccucccaugccaa....................  19   0    LCK-3
.........................................................................................................................................................................................................cugccuauuccucccaugccCa....................  1    1    JCK-1
.........................................................................................................................................................................................................cugccuauuccucccaugccaa....................  4    0    LT-3
.........................................................................................................................................................................................................cNgccuauuccucccaugccaa....................  1    1    JCK-2
.........................................................................................................................................................................................................cugccuauuccucccaugccaa....................  27   0    JCK-1
.........................................................................................................................................................................................................cugccuauuccucccaugccaU....................  1    1    LT-1
.........................................................................................................................................................................................................cugccuauuccucccaugccaC....................  1    1    JCK-2
.........................................................................................................................................................................................................cugccuauuccucccaugccaa....................  12   0    LT-1
.........................................................................................................................................................................................................cugccuauuccucccaugccaa....................  8    0    JT-1
.........................................................................................................................................................................................................cugccuauuccucccaugccaa....................  66   0    JCK-2
.........................................................................................................................................................................................................cugccuauuccucccaugccaUu...................  1    1    JCK-1
.........................................................................................................................................................................................................cugccuauuUcucccaugccaau...................  2    1    JCK-1
.........................................................................................................................................................................................................cugccuauuccucccaugccaau...................  1    0    JCK-2
.........................................................................................................................................................................................................cugccuauuccucccaugccaUu...................  2    1    LT-1
.........................................................................................................................................................................................................cugccuauuccucccaugccaUu...................  2    1    JCK-3
.........................................................................................................................................................................................................cugccuauuccucccaugccaaC...................  1    1    LT-1
.........................................................................................................................................................................................................cugccuauuccucccaugccaauU..................  1    1    JCK-3
.........................................................................................................................................................................................................cugccuauuccucccaugccaauU..................  1    1    JCK-2
.........................................................................................................................................................................................................cugccuauuccucccaugccaauU..................  1    1    LT-3
..........................................................................................................................................................................................................ugccuauuccucccaugc.......................  7    0    LCK-3
..........................................................................................................................................................................................................ugccuauuccucccaugc.......................  1    0    LT-2
..........................................................................................................................................................................................................ugccuauuccucccaugcc......................  2    0    LT-2
..........................................................................................................................................................................................................ugccuauuccucccaugcc......................  1    0    LT-1
..........................................................................................................................................................................................................ugccuauuccucccaugccU.....................  1    1    LT-1
..........................................................................................................................................................................................................ugccuauuccucccaugcca.....................  6    0    LT-1
..........................................................................................................................................................................................................ugccuauuccucccaugcca.....................  9    0    LCK-3
..........................................................................................................................................................................................................ugccuauuccucccaugcca.....................  3    0    JCK-2
..........................................................................................................................................................................................................ugccuauuccucccaugcca.....................  2    0    LT-3
..........................................................................................................................................................................................................ugccuauuccucccaugccaa....................  1    0    JCK-1
..........................................................................................................................................................................................................ugccuauuccucccaugccaa....................  4    0    JCK-2
..........................................................................................................................................................................................................ugccuauuccucccaugccaa....................  1    0    JCK-3
..........................................................................................................................................................................................................ugccuauuccucccaugccaau...................  1    0    LT-1
............................................................................................................................................................................................................ccuauuccucccaugcca.....................  1    0    LT-3
..............................................................................................................................................................................................................uauuccucccaugccaaugauuuc.............  1    0    JCK-3
..............................................................................................................................................................................................................uauuccucccaugccaaugauuuc.............  1    0    JCK-1
.................................................................................................................................................................................................................uccucccaugccaaugauuuc.............  1    0    JCK-3
.......................................................................................................................................................................................................................................ugauuuccuuuuguuccu..  1    0    LT-3
.......................................................................................................................................................................................................................................ugauuuccuuuuguuccu..  1    0    JCK-2
.......................................................................................................................................................................................................................................ugauuuccuuuuguuccuc.  3    0    JCK-1
.......................................................................................................................................................................................................................................ugauuuccuuuuguuccuc.  1    0    LT-1
.......................................................................................................................................................................................................................................ugauuuccuuuuguuccuc.  5    0    LT-2
.......................................................................................................................................................................................................................................ugauuuccuuuuguuccuc.  4    0    LT-3
.......................................................................................................................................................................................................................................ugauuuccuuuuguuccuc.  1    0    JCK-2
.......................................................................................................................................................................................................................................ugauuuccuuuuguuccucu  1    0    JCK-1
```

```
Star                                                                                                                                                              Mature
auaaccaucaaaagugaagacccaaugcauuaaauuaaucaauagugucuccuucuuuggacagcuccucuuaucgucauuuucaaggcugacaucaacgcguuuuaaagagagauaaagaacuuauggaaauuuuuggcuaugggagguuuggcaagaauaaaaauaauuauuuacaagauaaaaaaaaaaaaaaagcauguuuuuuccugccuauuccucccaugccaaugauuuccuuuuguuccucu
.......................................................................................................................................................................................................................................ugauuuccuuuuguuccucA    1      1      LT-2
.......................................................................................................................................................................................................................................ugauuuccuuuuguuccucu    1      0      JCK-3
.......................................................................................................................................................................................................................................ugauuuccuuuuguuccucu    1      0      LCK-3
```
